# α9-nicotinic acetylcholine receptors contribute to the maintenance of chronic mechanical hyperalgesia, but not thermal or mechanical allodynia

**DOI:** 10.1186/1744-8069-10-64

**Published:** 2014-10-02

**Authors:** Sarasa Mohammadi, Macdonald J Christie

**Affiliations:** Department of Pharmacology, The University of Sydney, Sydney, NSW Australia

**Keywords:** Nicotinic acetylcholine receptor, Pain, Chronic pain, Mechanical hyperalgesia, Allodynia

## Abstract

**Background:**

The current pharmacological treatments for chronic pain are limited. The first analgesic drug approved for clinical use in decades that has a novel molecular target is the synthetic version of a naturally occurring conotoxin. Several conotoxins that target ion channels have progressed to clinical trials for the relief of pain. Vc1.1 and RgIA are analgesic α-conotoxins that target α9-subunit-containing nicotinic acetylcholine receptors (α9-nAChR) as well as GABA_B_ receptor mechanisms. However, the evidence for the involvement of α9-nAChRs in pain is controversial. In the present study, the role of the α9-nAChR in pain was assessed using a battery of behavioural pain tests and pain models in α9-nAChR knockout (KO) mice.

**Results:**

α9-nAChR KO mice showed normal responses to acute noxious thermal and mechanical stimuli, and developed normal chronic cold and mechanical allodynia in inflammatory and nerve injury pain models. However, KO animals developed mechanical hyperalgesia to a lesser extent than their wild type (WT) counterparts in both inflammatory and neuropathic pain models. Chronic neuropathic pain is sustained in WT mice for at least 21 days post injury, while KO mice show significant recovery by 14 days post injury. KO sham mice were also resistant to the repeated-measures effect of the noxious pain test that caused a gradual onset of mild mechanical hyperalgesia in WT sham animals.

**Conclusions:**

The α9-nAChR is not involved in acute pain perception or chronic thermal or mechanical allodynia or thermal hyperalgesia but does contribute to the intensity and duration of chronic mechanical hyperalgesia, suggesting that pain-relieving actions of antagonists that target this site may be restricted to high threshold mechanosensation. The α9-nAChR appears to be a valid target for pharmacological compounds that alleviate long-term mechanical hyperalgesia and may be of use as a prophylactic drug to prevent the development of some symptoms of chronic pain.

Chronic pain is a significant worldwide health problem [1–3]. Current therapeutic options are inadequate for many pain sufferers as they act on very few molecular targets, commonly leading to insufficient relief, dose-limiting side effects and development of tolerance [4, 5]. Conotoxins are disulfide-rich peptides isolated from the venom of carnivorous marine cone snails [6]. The cocktail of peptides that constitute the venom include highly potent and specific ligands for many ion channels and receptors, providing a rich source of potential therapeutics for pain management. ω-Conotoxin MVIIA (Prialt®, ziconotide) was the first conotoxin approved by the FDA for management of chronic pain. Ziconotide, which inhibits pain transmission by inhibition of pre-synaptic N-type Ca^2+^ channels [7], has proven more efficacious than currently available analgesics in some individuals [8], and is not prone to tolerance or addiction. However, the use of ziconotide is very limited because it requires intrathecal administration and has a very narrow therapeutic window with potentially severe side effects [7, 9]. Other classes of conotoxins acting on novel targets may provide therapeutic advantages in pain management [6, 10].

Both nicotinic acetylcholine receptor (nAChR) agonists and antagonists have been implicated as analgesics. Whilst non-specific agonists of nAChRs have long been known to alleviate pain [11], targeting specific receptor subunit(s) or stoichiometries has met with limited success. Thus the precise mechanism of therapeutic action remains elusive [12–15]. Some α-conotoxins that are nAChR antagonists have been shown to reverse signs of chronic pain in animal models after systemic administration, e.g., Vc1.1 is an α-conotoxin that partially reverses mechanical allodynia and hyperalgesia in animal models of neuropathic pain [16–20]. Additionally, accelerated functional recovery from nerve injury is seen in Vc1.1-treated animals [17, 18]. However, the mechanism by which Vc1.1 achieves this action is controversial. Vc1.1 was first thought to relieve mechanical hyperalgesia by antagonism of nAChRs containing α3 subunits [16–18]. However the potency of Vc1.1 at α3β2, α3β4 and α3α5β2 nAChRs is rather weak [21]. More recently, it was identified that analgesic conotoxins Vc1.1 and RgIA are very potent antagonists of α9 subunit-containing nAChRs (α9-nAChR), suggesting that this subtype, not previously implicated in nociception, is the analgesic target [19]. It was speculated that the sustained reversal of mechanical hyperalgesia observed after Vc1.1 or RgIA-treatment are due to a reduced inflammatory response in the nerve injury model, with diminished numbers of immune cells and ACh-producing cells observed at the nerve injury sites of conotoxin treated animals [19]. Non-peptide, small molecule α9-nAChRs agonists have also been reported [22, 23]. Whilst they do not affect acute thermal nociception (tail-flick), they do reduce mechanical hypersensitivity in chronic pain models (CCI or vincristine) as well as attenuate the development of neuropathies (vincristine, phase II of formalin test). However, there is some doubt that α9-subunit containing nAChRs are the primary pharmacological target of α-conotoxins because several analogues of Vc1.1 that are equipotent with this peptide at α9α10-nAChRs were shown not to produce reversal of mechanical allodynia in a rat model of neuropathic pain [24].

A second potential target for several analgesic α-conotoxins, including Vc1.1, has been identified whereby N-type calcium channel currents are inhibited via a novel GABA_B_-receptor dependent mechanism [25]. The necessity of the GABA_B_ receptor in this mechanism has been shown *in vitro* and *in vivo*, with Vc1.1 shown to be ineffective both for inhibition of calcium channel currents and reversal of nerve-injury-induced mechanical allodynia in the presence of GABA_B_ receptor antagonists [20, 25, 26]. Moreover, analogues of Vc1.1 that retain potency for the novel GABA_B_ receptor mechanism but are very weak inhibitors of α9-nAChRs are analgesic [27].

To address the controversy the current study examined the behavioural phenotypes of α9-nAChR knockout (KO) mice compared with wild type (WT) mice in both acute and chronic pain models. Our results show a unique pain phenotype. Acute thermal and mechanical nociception is normal in α9-nAChR KO mice. In chronic pain models, α9-nAChR KO mice exhibit reduced development of chronic mechanical hyperalgesia but are indistinguishable from WT animals in the development of mechanical and cold allodynia, and thermal hyperalgesia.

## Results

### Acute nociception is unaffected by deletion of the α9-nAChR

**Figure 1:**
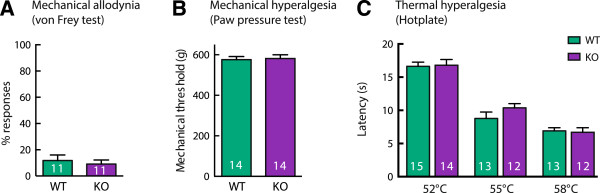
**Acute nociception is normal in α9-nAChR KO mice.** Acute tactile and mechanical nociceptive thresholds were measured in naïve mice using the von Frey test **(A)** and the paw pressure test **(B)** respectively. Acute thermal nociception was measured using the hotplate **(C)**.

Thermal and mechanical nociceptive thresholds in α9-nAChR KO mice did not differ significantly from WT counterparts. In naïve animals mechanical nociception was normal when tested using the von Frey (Figure 1A, t(20) = 0.52, P = 0.61) and paw pressure tests (Figure 1B, t(26) = 0.22, P = 0.82). Thermal nociception was also unaffected by the KO when tested at three noxious temperatures on the hotplate (Figure 1C, no significant genotype effect F(1,73) = 0.76, P = 0.39).

### Chronic hot and cold hypersensitivity is normal in α9-nAChR mice

**Figure 2:**
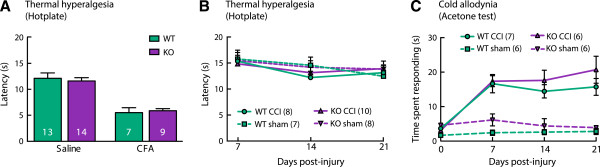
**Chronic hot/cold hypersensitivity is normal in α9-nAChR KO mice.** Thermal hyperalgesia was tested on a 54°C hotplate. Thermal hyperalgesia develops normally in both WT and α9-nAChR KO mice in an inflammatory pain model **(A)** and is characteristically absent in a neuropathic pain model **(B)**. Cold allodynia is present in both genotypes after CCI and is maintained for at least 3 weeks post-injury **(C)**.

Changes to both hot and cold sensory modalities post-injury were tested in models of inflammatory (Freund’s complete adjuvant; CFA) and neuropathic (chronic constriction injury; CCI model) pain. Thermal hyperalgesia was tested on the hotplate. The inflammatory pain model produced a decreased response latency at 4 days post injury (Figure 2A, significant treatment effect, F(1,39) = 52.16, P < 0.0001) that did not differ between WT and KO animals (no significant genotype effect, F(1,39) = 0.008, P = 0.93). The neuropathic pain model did not produce thermal hyperalgesia in either WT or KO animals (Figure 2B, no significant treatment effect, F(1,29) = 0.489, P = 0.49 or genotype effect F(1, 29) = 0.13, p = 0.72). Cold allodynia evoked by the acetone test was observed after CCI (Figure 2C, significant treatment effect, F(1,21) = 83.82, p < 0.0001) and did not differ between WT and KO animals (Figure 2C, no significant genotype effect, F(1, 21) = 4.1, P = 0.06).

### Chronic mechanical allodynia develops normally in α9-nAChR KO animals, but mechanical hyperalgesia shows a unique phenotype

**Figure 3:**
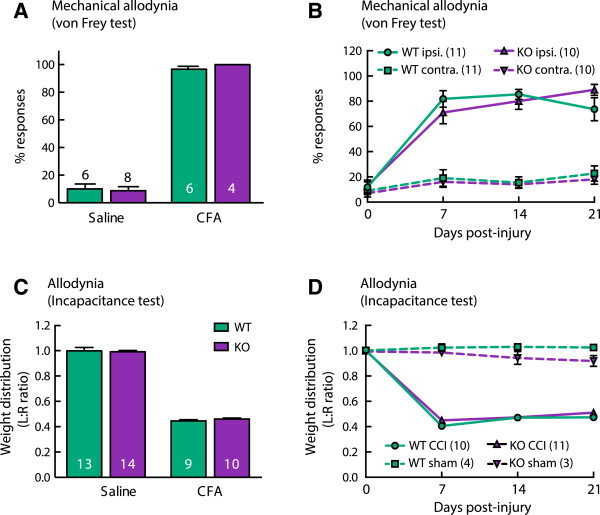
**Chronic mechanical allodynia develops normally in α9-nAChR KO mice.** Inflammatory **(A and C)** and neuropathic **(B and D)** pain models produce chronic mechanical allodynia in both WT and KO animals. Response frequency to von Frey stimulation increases following injury **(A and B)**. The ratio of weight-bearing on injured versus uninjured hind-paws decreases post injury **(B and C)**.

**Figure 4:**
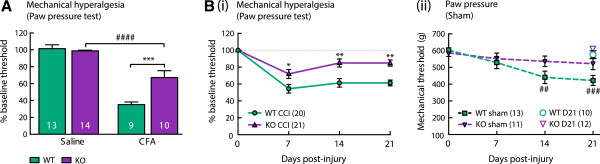
**Chronic mechanical hyperalgesia is reduced in α9-nAChR KO mice.** Inflammatory **(A)** and neuropathic **(B)** pain models produce chronic mechanical allodynia in both WT and KO animals. However, the magnitude of hyperalgesia is less in α9-nAChR KO animals. α9-nAChR KO animals recover from CCI by 14 days post injury **(B i)** and are resistant to the repeated-measures induced hyperalgesia of the paw pressure test seen in WT animals **(B ii)**. ##, p < 0.01; ###, p < 0.001; ####, p < 0.0001 compared to baseline. *, p < 0.01; **, p < 0.01; ***, p < 0.001 compared to WT.

Chronic mechanical allodynia developed normally in α9-nAChR KO mice. Inflammatory (Figure 3A and C) and neuropathic (Figure 3B and D) pain models both induced mechanical allodynia as expected. Maximal von Frey responses were reached 4 days after CFA injection (F(1,20) = 875.5, p < 0.0001) and 7 days after CCI (F(1,38) = 75.85, p < 0.0001) compared with control groups. Continuation of testing over a 21-day duration after CCI showed sustained allodynia that did not differ between WT and KO (no significant genotype effect, F(1, 38) = 0.13, P = 0.72), with sustained increased responsiveness to the von Frey test on the injured hind-paw, but not on the contralateral hind-paw over 21 days (Figure 3B). For the incapacitance test L:R ratio of weight bearing on the hind paws remained lowered after CCI but not sham surgery (Figure 3D) and did not differ between WT and KO over 21 days (no significant genotype effect, F(1,24) = 2.79, P = 0.10).Mechanical hyperalgesia developed in both genotypes, however the magnitude of hypersensitivity was less in α9-nAChR KO animals compared to WTs in both inflammatory (significant genotype effect, F(1,42) = 10.35, P = 0.003 using two-way ANOVA; multiple comparisons between WT and KO CFA, P < 0.001) (Figure 4A) and neuropathic (significant genotype effect F(1,39) = 18.14, P = 0.0001 using two-way ANOVA by treatment; multiple comparisons between WT and KO CCI, P = 0.05) (Figure 4B) pain models at 4 and 7 days post injury respectively. Continuation of testing weekly after CCI surgery showed that mechanical hyperalgesia persists in WT mice for at least 21 days (P < 0.0001 for each test day compared to normalised baseline, three-way ANOVA with multiple comparisons and Bonferroni post-hoc test), whereas the KO strain shows significant recovery by 14 days post injury (P < 0.0001 on D7, P < 0.05 at D14 and D21 compared to baseline) (Figure 4B i).The noxious nature of the mechanical stimulus used repeatedly in the paw pressure test induced a mild mechanical hyperalgesia in the sham-operated WT mice, apparent at day 14 (P < 0.01) and day 21 (P < 0.001, three-way ANOVA with multiple comparisons and Bonferroni post-hoc test) (Figure 4B ii). The α9-nAChR KO mouse strain was resistant to this repeated-testing effect (no significant change from baseline for KO animals on all post-injury test days, P > 0.05, three-way ANOVA with multiple comparisons). The decrease in threshold of WT sham mice was not due to a delayed effect of the sham surgery, as separate groups of sham-operated WT and KO mice tested only on post-injury day 21 did not differ and had thresholds comparable to raw baseline scores of sham animals (F(3,42) = 0.98, P = 0.41, one-way ANOVA).

## Discussion

The discovery of analgesic α-conotoxins, such as Vc1.1 and RgIA, initiated interest in the α9-nAChR as a potential target for pain relief. This study has investigated the behavioural effect of α9-nAChR deletion. We found that α9-nAChR KO mice display a largely normal pain phenotype, with acute nociception and chronic allodynia indistinguishable from WT mice. Differences were detected only in the domain of chronic mechanical hyperalgesia, wherein α9-nAChR KO mice are more resistant to the development of mechanical hyperalgesia, and this lessened hyperalgesia resolves more rapidly than in the WT counterparts.

Clearly, the absence of functional α9-nAChR subunits does not abolish nociception, and does not account for the documented acute anti-allodynic [20, 24, 27, 28] and anti-hyperalgesic [16, 18, 19, 28] effects of α-conotoxins. Studies on Vc1.1 and RgIA continue to demonstrate beneficial effects of these conotoxins in preclinical models, and authors continue to attribute their mechanisms of action to α9-nAChR inhibition [29]. The present study demonstrates that the clinical relevance of α9-nAChR inhibition may be limited to a specific modality of chronic pain, wherein mechanical hyperalgesia may be prevented and alleviated without benefit in other modalities such as light touch (allodynia) or thermal pain. We hypothesise that certain analgesic α-conotoxins, such as Vc1.1, RgIA and AuIB, operate via a dual mechanism of action, whereby both nAChR and N-type Ca^2+^ channels are inhibited [20, 26, 30, 31]. Inhibition of the α9-nAChR may be conferring an additional attenuating and restorative capacity to the conotoxins, alongside the GABA_B_-dependent N-type Ca^2+^ channel inhibition [20, 25].

We observed dissociation between mechanical allodynia and hyperalgesia in the manifestation of pain in α9-nAChR KO mice. This dissociation may have been masked in α-conotoxin studies due to the dual mechanism of action, while Vc1.1-analogues that specifically target α9-nAChR but not the GABA_B_-receptor-dependent mechanism [25] were shown to have no acute analgesic effects in animal models [24]. Some studies have reported specific pharmacological block of the α9-nAChR with quaternary ammonium analogues of nicotine, finding those compounds to be effective at attenuating the development of vincristine-induced pain (von Frey and paw pressure vocalisation threshold) and phase II formalin-pain, as well as acutely relieving CCI- and vincristine-pain (paw pressure vocalisation threshold) at high doses [22, 23]. It remains possible that off-target effects of these small molecule antagonists explain the discrepancy with the very selective effect on mechanical hyperalgesia observed in the present study. In summary, the results presented here suggest a sensory neuron type specific effect of α9-nAChR analgesia, where high threshold mechanoresponsive C fibres [32] are likely to be involved only under conditions of chronic inflammatory or neuropathic pain.

The mechanism responsible for this selective disruption of mechanical hyperalgesia by deletion of the α9-nAChR is unknown. The anatomical site of the α9-nAChR’s involvement in pain remains uncertain. The majority of studies investigating this receptor subtype have been in audition and the cochlea. In pain-related cell types, either an absence of expression is reported or findings are conflicting. The α9-nAChR is not expressed in the CNS [33]. In the periphery, expression in sensory afferent axons appears to be absent [33, 34], while expression in DRGs has been shown at various levels of the spinal cord. However, studies of expression in DRGs is limited to mRNA detection [34, 35], and α9-nAChR protein has not been identified via immunohistochemical methods [25]. Inhibition of immune cell activity, presumably inhibiting the downstream release of inflammatory and algogenic molecules, has been suggested as a potential target of α9 nAChR antagonists [19]. A reduction in immune cell infiltration of injured sciatic nerves was shown in Vc1.1-treated animals compared to controls, which was attributed by the authors to α9-nAChR inhibition. It would be interesting to investigate any such difference in α9-nAChR KO mice compared to WTs. In the present study, the CFA model of inflammatory pain did show significantly less hyperalgesia in KOs than WTs, however, Vc1.1 has shown only weak activity in this model [28]. Studies have shown expression of α9-nAChR RNA in human blood lymphocytes [36] and protein in mouse splenic B-lymphocytes [37], however no demonstrable functional α9-nAChR response to ACh has been achieved in lymphocytes [36].

An alternative possibility for the mechanism of α9-nAChR antagonism in mechanical hyperalgesia is the neuroendocrine system. The widely varying aetiologies of neuropathy (neuropathic [16, 18–20, 24, 27], inflammatory [28], chemogenic [23], metabolic [28]) that benefit from α9-nAChR inhibition might suggest a higher-order system effect. The α9-nAChR is expressed in the anterior pituitary and adrenal glands [33], and is upregulated in the adrenal gland in cold-stressed animals [38]. Hypothalamic-pituitary-adrenal (HPA) axis dysregulation is associated with chronic pain [39, 40], and α9-nAChR activity in the HPA-axis might contribute to the enhanced and sustained pain by affecting changes in circulating stress hormones such as corticotrophin, and catecholamines. It would be of interest to determine in future studies whether HPA-axis regulation is affected in the α9-nACh KO mice.

Deleterious side-effects of α9-nAChR-inhibiting contoxins have not been reported. α9-nAChRs are expressed in a range of tissues, which may make α9-nAChR-inhibiting therapeutics vulnerable to side-effects. The α9-nAChR is best understood in the auditory system, where it is involved in efferent auditory feedback. Inhibition of auditory α9-nAChRs may cause predisposition to noise-induced hearing loss [41]. This would only be expected for drugs that cross the blood-cochlear barrier [42] which is unlikely for peptide conotoxins. CNS side effects, such as memory disruption produced by drugs targeting other nAChRs would not be expected of highly selective α9-nAChR inhibitors because there is no evidence for expression of this subtype in CNS [33]. In the periphery, α9-nAChRs also play a role in epithelial cell proliferation, and are up-regulated in some cancers (breast [43] and lung [44]). Side effects in epithelial cell regulation might therefore be produced. Expression in the HPA axis [33, 38] may also impart α9-inhibitors with susceptibility to mood- or endocrine side-effects.

The present study suggests that the inhibition of the α9-nAChR has been erroneously attributed to be the mechanism of acute α-conotoxin analgesia. Such misattribution may be due to the unusual properties of the α9-nAChR, leading to misinterpretation of data. Nicotine, an α9-nAChR antagonist, has been routinely used as an agonist in α-conotoxin specificity and efficacy studies [17, 21, 27, 45, 46]. Such assays continue to be used in the context of Vc1.1 analgesia [27], however the nAChR subunit target in these assays is unknown (for a list of known nAChR combinations, see Millar & Gotti, [47]). In many of these assays [17, 21, 27, 46], the α9-nAChR may have been rendered inactive in the tissue preparation process, as collagenase, the primary digestive enzyme used, uncouples the α9-nAChR from Ca^2+^-dependent K^+^ channel (SK2) which is a complex shown to be necessary for function [48]. However, it is possible that the requirement of α9-nAChR/SK2 coupling is specific to the vestibular hair cell type, since functional α9-nAChRs have been recombinantly expressed in *X. laevis* oocytes [19, 21, 24, 27, 30, 49].

## Conclusions

Germline deletion of the α9 subunit of nACh receptors produces an unusual pain phenotype in mice. Thermal hyperalgesia is unaffected and both thermal and mechanical allodynia develop normally. By contrast, mechanical hyperalgesia is attenuated and recovers more rapidly in KO mice. Although the α9-nAChR was first implicated in pain as the mechanism of action of some analgesic α-conotoxins, the present study shows that inhibition of this receptor alone cannot account for the analgesic effects of Vc1.1 and RgIA. α9-nAChRs may be a valid target for pharmacological compounds that alleviate long-term mechanical hyperalgesia, perhaps via promoting recovery. The precise mechanism and anatomical location of the α9-nAChRs involved remains to be determined.

## Materials and methods

### Animals

All experiments involving animals were approved by the University of Sydney Animal Ethics Committee. Experiments were performed under the guidelines of the Australian code of practice for the care and use of animals for scientific purposes (National Health and Medical Research Council, Australia, 7th Edition). Great care was taken to minimise animal suffering during these experiments whenever possible. In vivo experiments were performed on 380*,* 6–8 week old male 129Sv/Ae mice. Mice were housed no more than six per cage and were maintained on standard 12 hour light/dark cycle with free access to food and water. α9-nAChR knockout (KO) mice were obtained from Dr. Douglas Vetter (Tufts Univ, Boston MA). Receptor deletion was confirmed by genotyping using standard PCR procedures.

### Induction of chronic pain

#### Neuropathic pain

A chronic constriction of the sciatic nerve was applied using a cuff (n = 93), in a method adapted from Mosconi & Kruger [50] and Benbouzid et al. [51]. Briefly, the common branch of the left hind-limb sciatic nerve was exposed and a 2 mm section of PE-20 polyethylene tubing (Clay Adams® BD & Co. Maryland, inner diameter 0.38 mm, outer diameter 1.09 mm) split longitudinally was placed around it. Sham operated mice (n = 80) underwent the same surgical procedure, omitting the implantation of the cuff.

#### Inflammatory pain

To induce a unilateral chronic inflammatory pain state, 50 μL of complete Freund’s adjuvant (CFA) (Sigma-Aldrich, USA) was injected subcutaneously into the plantar surface of the left hind-paw (n = 45). Control mice underwent the same procedure, however, saline was injected in place of CFA (n = 68). Injections were performed using 29G needles. All surgical procedures were carried out under isoflurane anaesthesia (2.0-2.5% in oxygen). No paw drooping or autotomy was observed in any of the nerve injured or CFA-injected mice.

### Behavioural tests

Behavioural tests of pain thresholds were performed at time-points that allowed sufficient recovery from the injury-procedures, as well as allowing maximal pain thresholds to be reached. The progression of neuropathic pain was measured at weekly intervals for a maximum of three weeks post-injury.

#### Thermal hyperalgesia

Thermal hyperalgesia was assessed using the hot-plate test. With minimal animal-handler interaction, mice were taken from home-cages, and placed onto the surface of the hot-plate (IITC Life Sciences) maintained at a constant, noxious temperature (52–58 ± 0.2°C as shown in Figure 1). Ambulation was restricted by a cylindrical Plexiglas chamber (diameter: 10 cm, height: 15 cm), with open top. A timer controlled by foot peddle began timing response latency from the moment the mouse was placed onto the hot-plate, and was stopped upon the first behaviour indicative of nociception. Endpoints that terminated the test were hind-paw shake or lick, and jumping. Mice were immediately removed from the hotplate.

#### Cold allodynia

Cold allodynia was assessed by measuring the acute nocifensive responses to acetone-evoked evaporative cooling [52, 53]. For 2 days prior to testing, and on testing days, mice were habituated in mesh-floor testing chambers for at least 1 hour. To test, 10 μL of acetone (analytical grade, Bacto) was propelled onto the plantar surface of the left hind-paw. The air burst from a 100 μL pipette was used to project the acetone, thus avoiding mechanical stimulation of the paw with the pipette tip. The time spent lifting, licking or shaking the hind-paw over a 60 s time period was recorded. Acetone was applied 3 times with at least 5 min intervals.

#### Mechanical incapacitance test

Differences in static weight distribution across hind paws was assessed using a dual channel weight averager, the Linton Incapacitance Tester (MJS Technology Ltd., Hertfordshire, UK), as described previously in rats [54] and mice [55]. Mice were placed at the opening of a transparent acrylic chamber, which they freely enter. The chamber design encourages an upright posture, with the majority of weight placed on the hind-paws, and forepaws resting on an inclined plane, for balance and support. The floor of the chamber is split equally into two electronic weighing scales, and measurements are taken when each hind-paw rests symmetrically on each scale. The average of six measurements were taken and represented as a ratio of ipsilateral (left hind-paw) to contralateral (right hind-paw) weight distribution. Decreased ratio indicates less weight placed on the injured hind-paw, and is herein referred to as mechanical allodynia.

#### Von Frey filaments

The von Frey test was used to assess the development of mechanical allodynia. For 2 days prior to testing and on testing days, mice were habituated in testing chambers for 1 hour. In order to determine mechanical allodynia, a calibrated von Frey filament (Stoelting Co. Chicago, IL) was pressed perpendicularly to the plantar surface of both ipsilateral and contralateral mouse hind-paws, until the filament just buckled. A single grade of filament of size 3.61 was chosen, that elicited mean response frequencies of approximately 15% at baseline. The hair was applied 10 times for a duration of 5 sec, with an interval of at least 1 min between each stimulation. Responses of brisk withdrawal (flinches, vigorous shaking/kicking) and licking were considered as positive nociceptive responses. Scores are represented as a percentage of positive responses.

#### Paw pressure test

Mechanical nociceptive thresholds were measured using a Pressure Application Measurement (PAM) analgesymeter with Paw Pressure Transducer (Ugo Basile). Analogous to the Randall-Siletto test for rats [56], mice were gently restrained and a mechanical pressure was applied to their left hind-paw. The plantar surface is supported against the flat base of the paw pressure transducer, while the blunt conical probe is pressed against the dorsal surface at a linearly increasing force (maximum 600 g). The force at which paw withdrawal is elicited is automatically recorded by the PAM device. The peak forces of 3 trials were averaged for each mouse, with at least 5 min inter-trial interval.

### Expression of data and statistical analyses

Data were analysed using Prism (GraphPad Software Inc. version 6.0b for Mac OS X, San Diego, CA, USA) and SPSS (IBM® SPSS® Statistics, Version 21, Armonk, NY) software. All data are presented as mean ± SEM.

Student’s t-tests and one-, two- and three-way ANOVAs were performed using Prism software. The between-subjects main effects of genotype (WT vs. KO) and treatment (temperatures or injury) were evaluated with two-way ANOVAs. Within-subjects effects (days post-injury) were analysed with three-way ANOVAs comparing genotype and treatment groups with a fixed measure (normalised baseline score). When significant effects were observed, Bonferroni’s post-hoc tests were used. Time-course data were analyzed using SPSS general linear model with repeated measures, with time as a within subjects factor. P < 0.05 was considered significant. Significant effects are shown throughout as * = P < 0.05, ** = P < 0.01, *** = P < 0.001, **** = P < 0.0001.

## Abbreviations

CFA: Freund’s complete adjuvant
CCI: Chronic constriction injury
DRG: Dorsal root ganglion
HPA: Hypothalamic pituitary adrenal
nAChR: nicotinic acetylcholine receptor
SK2: Small conductance calcium-dependent potassium channel.
